# The Effect of Task-Irrelevant Fearful-Face Distractor on Working Memory Processing in Mild Cognitive Impairment versus Healthy Controls: An Exploratory fMRI Study in Female Participants

**DOI:** 10.1155/2016/1637392

**Published:** 2016-02-01

**Authors:** Amer M. Burhan, Udunna C. Anazodo, Jun Ku Chung, Amanda Arena, Ariel Graff-Guerrero, Derek G. V. Mitchell

**Affiliations:** ^1^Division of Geriatric Psychiatry at Schulich School of Medicine, Parkwood Institute, Mental Health Care Building, F2-349, London, ON, Canada N6C 0A7; ^2^Lawson Imaging, Lawson Health Research Institute, London, ON, Canada N6A 4V2; ^3^CAMH, Toronto, ON, Canada M5T 1R8; ^4^Department of Psychiatry at Schulich School of Medicine, The Brain and Mind Institute, Natural Sciences Centre, London, ON, Canada N6A 5B7

## Abstract

In mild cognitive impairment (MCI), a risk state for Alzheimer's disease, patients have objective cognitive deficits with relatively preserved functioning. fMRI studies have identified anomalies during working memory (WM) processing in individuals with MCI. The effect of task-irrelevant emotional face distractor on WM processing in MCI remains unclear. We aim to explore the impact of fearful-face task-irrelevant distractor on WM processing in MCI using fMRI.* Hypothesis*. Compared to healthy controls (HC), MCI patients will show significantly higher BOLD signal in* a priori* identified regions of interest (ROIs) during a WM task with a task-irrelevant emotional face distractor.* Methods*. 9 right-handed female participants with MCI and 12 matched HC performed a WM task with standardized task-irrelevant fearful versus neutral face distractors randomized and counterbalanced across WM trials. MRI images were acquired during the WM task and BOLD signal was analyzed using statistical parametric mapping (SPM) to identify signal patterns during the task response phase.* Results*. Task-irrelevant fearful-face distractor resulted in higher activation in the amygdala, anterior cingulate, and frontal areas, in MCI participants compared to HC.* Conclusions*. This exploratory study suggests altered WM processing as a result of fearful-face distractor in MCI.

## 1. Introduction

*(1) Background*. Mild cognitive impairment (MCI) is a transitional risk state between cognitive aging and Alzheimer's disease (AD). This state is defined as a “cognitive concern,” with patients demonstrating deficits in one or more cognitive domains on objective testing while maintaining relatively preserved daily functioning. Individuals meeting criteria for MCI are at a higher risk of converting to AD compared to healthy controls [1]. In addition to cognitive deficits, patients with MCI often present with significant emotional symptoms including dysphoria, anxiety, and irritability [2]; these symptoms are associated with an increased risk for conversion from MCI to dementia and AD [3]. Furthermore, a recent study suggests that high level anxiety is a predictor of cognitive decline in a preclinical sample positive for beta-amyloid, a marker for AD risk [4].

Several lines of research have converged to better define MCI using clinical and biological markers. For instance, neuropsychological testing has identified a marked impairment in episodic memory, which is a defining clinical feature of this prodromal state [5] and can be identified up to 7 years before the onset of the diagnosis of AD [6]. Functional magnetic resonance imaging (fMRI), a modality that provides insight into brain activation patterns by comparing blood oxygen levels (BOLD) in brain areas of interest [7], has been used extensively to study cognitive disorders including MCI and AD. The preponderance of this work has focused on memory encoding tasks. These studies have yielded mixed results due to variability in several factors including the clinical definition of MCI, fMRI methodologies, cognitive task employed, and performance criteria (for a review, see [8]).

The effect of MCI on* other* cognitive domains including working memory (WM) has received less attention; however, growing evidence has emphasized the importance of developing a comprehensive disease profile for MCI [9]. For example, Tabert et al. (2006) found that MCI patients with deficits in other cognitive domains in addition to memory impairments were at a higher risk of conversion to AD [10]. Even patients classified as amnestic MCI (aMCI) were found to have deficits in at least one of five subdomains of executive function (i.e., divided attention, WM, inhibitory control, verbal fluency, and planning), with the most common deficit apparent for inhibitory control [11]. These findings highlight the importance of establishing a comprehensive neuropsychological profile of MCI and, as argued by some, further examination of WM impairments for the prediction of conversion to AD is required [12, 13].

There is also evidence to suggest that MCI is associated with functional abnormalities in WM networks. Functional connectivity networks have been shown to be impaired in MCI patients during a WM task using Magnetoencephalogram (MEG) technology [14]. Using fMRI technology, Bokde et al. (2010) studied patterns of brain activation in MCI patients and healthy controls during a WM task; compared to HC, MCI patients show altered patterns of brain activation during verbal WM tasks. Specifically, MCI patients showed higher frontal and parietal activation during the maintenance phase of a delayed match-to-sample task performed in the scanner. This suggested a compensatory mechanism to maintain the task in the MCI group [15]. Recruitment of brain regions including prefrontal and parietal areas to compensate for limited attentional resources during a high demand task has been reported in older adults. This is mainly evident when task performance is successful [16–18], but not when performance fails, indicating inefficient compensation with increased task difficulty [19]. This issue was investigated in the MCI state; Kochan et al. (2011) found the level of WM difficulty had a differential effect on brain activation patterns. Lower difficulty WM tasks result in overactivation in the right anterior cingulate and right precuneus, while higher difficulty tasks result in reduced activation in these areas and “deactivation” in the posterior cingulate-medial precuneus [20]. Furthermore, this differential pattern of brain activation in response to a graded WM challenge predicted functional decline in MCI patients [21].

The interaction between emotional and cognitive processing has received significant attention in psychological literature. This issue was extensively reviewed by Dolcos et al. (2015) in a special research topics edition in Frontiers in Neuroscience and Frontiers in Psychology [22]. While emotional information can enhance episodic memory formation [23, 24], and WM [25, 26], task-irrelevant emotional interference has been shown to impair WM [27–30]. Neuroimaging studies have also documented the interaction between emotional and attentional processing streams in the brain (for reviews, see [27, 28, 31]). Generally, there are two streams of activities to consider when it comes to the interaction between attention and emotion: a “hot” emotional bottom-up stream involving ventral neuronal systems including the amygdala and hippocampal formation and a “cold” executive top-down stream involving prefrontal and parietal cortices. The dissociation of these streams is thought to be the mechanism for WM impairment with emotional interference [32]. Another area that has been implicated is the anterior cingulate cortex (ACC), which has been associated with emotion-cognition integration. The dorsal ACC (midportion) is engaged mainly in cognitive conflict resolution, while more rostral ACC has a role in* emotional* conflict resolution [33–35]. There is evidence that performing an attentional task while being exposed to an emotional signal engages rostral ACC, superior parietal areas, and the lateral prefrontal cortex [36–38]. Recently, Ariza et al. (2015) demonstrated that older adults were less able to reorganize network topology when dealing with interference compared to younger adults [39]. In amnestic patients, interference has been shown to affect memory encoding [40]. Therefore, the introduction of emotional interference during a WM task is another way of competing for attentional resources.

Brain mechanisms involved in the processing of emotional interference during WM tasks have received little attention in the aging-cognition literature. In this regard, Döhnel et al. (2008) conducted an fMRI study to examine the effects of emotional stimuli on WM in male and female patients with MCI. Relative to HC, MCI patients demonstrated a negativity bias on the behavioral task [42]. Specifically, MCI patients had better recall for negative targets. The fMRI results also pointed to a difference between the two populations; MCI patients exhibited increased activity in the right precuneus for negative targets compared to HC. These differences were proposed to be indicative of compensatory mechanisms in the MCI group. This study used pictures from the International Affective Picture System (IAPS, [42]) as “targets” for the 2-back WM task and classified the pictures into neutral, negative, and positive emotional valence [41]. Very recently, Berger et al. (2015) investigated the effect of task-irrelevant emotional interference on WM processes in a cohort of mixed MCI and AD patients compared to HC. They used IAPS where positive, neutral, and negative valence pictures were presented as distractors (interference) during an *n*-back WM paradigm. Their sample included 12 mixed AD and MCI patients (both males and female) as compared to 12 HC. The authors reported higher activation in left prefrontal areas and the amygdala, as well as reduced cerebellar activation with increased task difficulty in AD/MCI group compared to HC. The HC group showed more widely distributed network of activation compared to AD/MCI. These findings led the authors to conclude that there must be a compensatory mechanism involving the prefrontal cortex and amygdala and dysfunctional inhibition of irrelevant distractor in the AD/MCI group [43]. Patients with MCI have difficulty distinguishing different emotional expressions like anger, fear, or sadness despite preserved facial recognition skills. For example, a study by Spoletini et al. (2008) has shown that patients at early stages of cognitive impairment such as amnestic MCI are significantly impaired in labeling low-intensity fearful-face pictures compared to healthy controls, while those with AD have impairment across different emotions and at high and low intensity [44]. Impairment in recognizing different emotional expression likely affects social functioning (see [45] for review). Brain areas involved in emotional face processing include the amygdala, hippocampus, and surrounding cortex [46, 47]. Despite ongoing debate in the literature, several lesion studies demonstrated the role of the amygdala in processing fear/threat related facial expression [48–52] and in functional imaging studies, fearful facial expression was found to activate the amygdala more reliably than other emotions [53, 54].

In summary, there is evidence for an interaction between WM and emotional processing whereby emotional stimuli can compete for attentional resources and interfere with the task at hand. MCI patients have impairments in WM processing, with a suggestion of compensatory brain mechanisms that tends to fail as task difficulty increases and with interference. There is also evidence of impaired emotional processing in MCI patients as demonstrated by the high rate of emotional symptoms, especially anxiety [55–57], and the emerging literature demonstrating a specific interaction between anxiety and measures of executive function in this population [58]. Of particular relevance, anxiety symptoms in MCI have important prognostic implications as they increase the risk for conversion to Alzheimer's disease and other types of dementia [59, 60].

Both studies that investigated this issue used *n*-back WM paradigm and IAPS database where they presented positive, neutral, and negative valence pictures. The study by Döhnel et al. (2008) used emotional picture as target for the task [42] while the study by Berger et al. (2015) used emotional pictures as task-irrelevant interference [41].

*(2) Objective and Hypotheses*. In this study, we aim to investigate the effect of task-irrelevant emotional face interference on WM brain processing in MCI patients. This is an important issue given the evidence of impaired WM and emotional face processing in this population and the effect of both on executive and social functioning and the risk for conversion to AD. As described above, fearful face has the highest potential to engage the amygdala, which exerts bottom-up regulation on the emotional-cognitive interaction. Therefore, for this investigation, we use standardized fearful-face stimuli as task-irrelevant distractors in a delayed response-stimulus match WM task. In this study, we used fearful facial expression because we are specifically interested in the role of the amygdala during our working memory task.

A study by Iordan et al. (2013) identified differential activation pattern in females compared to males in response to emotional stimuli whereby negative emotions trigger more “hot” bottom-up activation in females compared to males [61]. Other studies reported sex-related differences in functional connectivity of the amygdala at rest and during affective processing [62–64]. In order to reduce variability in brain activation pattern in our sample, we limited our study to female patients with MCI.

We hypothesized that presenting fearful-face distractors in the context of a WM task would increase the burden on attentional resources by imposing bottom-up competition for these resources. This would likely result in the engagement of compensatory mechanisms in regions of interest (ROIs) relevant to emotion-cognition integration in patients with MCI. Given the evidence that rostral anterior cingulate cortex (rACC) is involved in regulating emotional distraction during WM tasks [36, 37] and in dual-task conditions [65], we predicted that patients with MCI would show increased activity in this area relative to HC. Another area that has been reported to be involved in processing emotional distractors is the superior parietal cortex [37], which we predicted would also show increased activity in MCI patients. Furthermore, on the basis that the ventral and lateral prefrontal cortices are associated with WM performance and attentional control [38, 66], we predicted that patients with MCI would show increased activity in these areas compared to HC.

## 2. Materials and Methods

### 2.1. Participants

Twenty-two participants were included in the study (HC = 12; MCI = 10). All subjects were right-handed females.  Table 1 summarizes demographic and clinical data. There was no significant difference in age between the two groups though MCI participants trended to be older. MCI participants were recruited from the memory clinic of an academic hospital in London, Ontario. They were referred to the clinic by family physicians on the basis of memory concerns. A diagnosis of MCI was confirmed using the Peterson criteria [68]. The clinic also administered the Clinical Dementia Rating (CDR) Scale; the CDR is a semistructured clinical interview that includes the patient and a reliable informant (typically a family member), to confirm the existence of a significant memory change from baseline with preservation of other aspects of cognitive function including language, visual spatial function, and executive function [67]. Two other commonly used clinical scales were used to assess symptoms of depression and cognitive function in participants: the Geriatric Depression Scale (GDS; [69]) and the Montreal Cognitive Assessment (MoCA; [70]) test, respectively. Based on the clinical assessment, CDR scale, and memory index >7/15 on MoCA test [71], these participants were classified as “amnestic” MCI though detailed neuropsychological testing was not available to confirm whether they were single- or multidomain “amnestic” MCI. Participants did not suffer any other psychiatric symptoms such as anxiety or psychosis as determined during the clinical interview by the memory clinic staff.

We excluded participants with neurodegenerative illnesses (such as any form of dementia or Parkinson's disease), stroke, traumatic brain injury (TBI), epilepsy, or major mental illness such as major depressive disorder (MDD), bipolar disorder, schizophrenia, or substance use disorder. None of the participants were taking cognitive enhancers. One MCI participant and one HC were taking a stable dose of a selective serotonin reuptake inhibitor for minor depressive symptoms. This study was approved by University of Western Ontario Research Ethics Board (HSREB #13178) and is in accordance with the tenets of the Declaration of Helsinki. Informed consent was obtained from all participants by study principle investigator (AMB) after providing a detailed letter of information and answering all participants' questions.

### 2.2. Stimuli and Tasks

**Figure 1:**
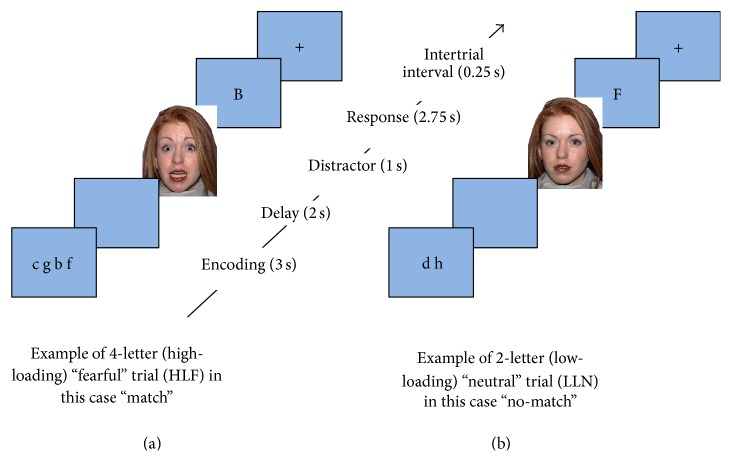
Structure of each “trial” including encoding phase, delay, fearful or neutral face distractor, response phase, and an intertrial interval. (a) illustrates a “match” high-loading, 4-letter trial with a fearful-face distractor. (b) illustrates a “no-match” low-loading, 2-letter trial with a neutral face distractor.

To test the impact of emotional stimuli on working memory performance, participants completed a novel visual WM task with emotional distractors. The WM task was designed using E-prime® 2 (Psychology Software Tools, Inc.) and each trial consisted of three components: an encoding phase, an interference phase, and a response phase (see Figure 1). The encoding phase of each trial was 3.0 sec long and consisted of a string of letters randomly selected from the first half of the alphabet and projected onscreen inside the scanner. Each participant was exposed to a total of 56 letters (50 consonants and 6 vowels). Letter strings were identical among all participants but their presentation was randomized and counterbalanced. After each encoding phase, a blank screen was presented for 2.0 sec to serve as a delay phase. The interference phase was 1.0 sec long and consisted of a task-irrelevant picture of a face presented on the screen. There were two different pictures for the interference phase: one face depicted a neutral expression (*N*) and the other a fearful expression (*F*). The facial images used in this study were selected from the standardized NimStim Face Stimulus Set after obtaining consent from Macbrain (http://www.macbrain.org/resources.htm). Immediately following the interference phase, one letter appeared onscreen in upper case and participants were asked to decide whether this letter was present in the string of letters previously shown. The rationale for presenting the letter in a different case was to increase the likelihood that participants would attend to the categorical identity of the letter when matching rather than the visual characteristics of the shape. For example, “b” and “B” will be a match despite different visual representation [72]. The response phase was 2.75 seconds long and subjects were asked to make a yes/no decision. The subjects pressed a button with the index finger of the dominant hand for positive answers and pressed the adjacent button with the middle finger of the same hand for negative answers. After the response phase, there was an intertrial interval of 0.25 seconds during which a fixation cross was present in the center of the screen. The total duration of each trial was 9 sec. All participants received a practice run on the paradigm before starting data acquisition to assure familiarity with the procedure.

Each subject performed two runs of the WM task, with each run composed of four blocks (7 trials/block, totaling 28 trials per run and 56 trials in total). As such, each run was 5.15 min (i.e., 309 sec) in duration. Each block presented one of the following four conditions: high-load (4 letters presented during the encoding phase) word stimuli with a fearful distractor (HF), low-load (2 letters presented during the encoding phase) word stimuli with a fearful distractor (LF), high-loading word stimuli with a neutral distractor (HN), and low-loading word stimuli with a neutral distractor (LN). The purpose of presenting high- and low-loading blocks was to assure adequate performance of participants on the task. The block order was counterbalanced and the interblock interval was 18 seconds long, during which a fixation cross was presented onscreen; this allowed the BOLD signal to return to baseline before commencing the next block. The response phase is where WM and face distractor are more likely to intersect. Therefore, BOLD signal was averaged across the response phase of trials carrying the same loading and emotional valance. Participants were given clear instructions at the beginning of each block with a sample task to assure clarity.

### 2.3. Scanning

Images were acquired on a 3-Tesla MAGNETOM Trio Tim (Siemens Medical Solutions USA, Inc.) whole-body scanner with a high-resolution 32-channel head coil. The participant's head was positioned along the canthomeatal line and immobilized by means of a forehead strap. T1-weighted sagittal images were used to select 54 contiguous oblique axial slices parallel to the anterior-posterior commissures plane. During the session, 103 volumes (54 slices each) covering the whole brain were acquired using a T2^*∗*^-sensitive EPI sequence (TE = 30 ms; TR = 3000 ms; flip = 90 degrees; image size = 640 × 640; slice thickness = 1.90 mm).

### 2.4. Data Analysis

Behavioral data were analyzed by way of a 2 (group: MCI or HC) × 2 (emotion: fearful or neutral) × 2 (high-loading or low-loading) mixed model Analysis of Variance (ANOVA) on response accuracy and reaction time (RT).

Image preprocessing and analysis were performed using statistical parametric mapping SPM8 (Wellcome Trust Centre for Neuroimaging, University College London; http://www.fil.ion.ucl.ac.uk/spm/). First, the scans for the first 6 sec were deleted to remove the initial T1 magnetic transients. Consequently, the images were transformed to analysis format for their further automatic realignment with SPM8 (6-parameter rigid body). During this procedure, 6 linear regressors were obtained describing the correction parameters applied at each volume. Data from subjects who showed movement of greater than two voxels on any axis were discarded. Subsequently, images were corrected for differences in the acquisition time of each slice within the same volume and spatially normalized to the EPI image template in SPM from the Montreal Neurological Institute (MNI). The voxel size was interpolated to 2 × 2 × 2 mm. The normalized images were smoothed with a Gaussian filter in each coordinate direction with a kernel of 8 mm. Individual analysis (first level) was performed with a general linear model (GLM) including the 6 rigid body correction parameter regressors as covariates in the design matrix.

T-contrast was performed for the response phase (event-related design) between neutral facial expressions and fearful facial expressions for each subject (first-level analysis) during the low-loading and high-loading task condition. Only the correct responses were included in the analysis, and the same numbers of correct trials were matched among subjects. The contrast images from each subject were employed for the second-level analysis.

We conducted a region-of-interest analysis between groups using a group × emotion × loading mixed model ANOVA and anatomically specified ROIs (bilateral anterior cingulate, prefrontal cortex, superior parietal, and amygdala areas) controlling for differences in age. Age was included as a covariate of no interest due to trend difference between groups. Loading and emotion were treated as within-subjects factors, while diagnosis (group) was treated as between-subjects factor. Anatomical ROI masks were generated using the WFU PickAtlas toolbox, version 3.0 [73], by combining the discrete regions. Across-group random effects of loading and between-group interactions of loading and emotion were explored voxel by voxel within the ROI mask to assess the effect of task difficulty. A cluster-level statistical threshold was set across all ROIs at *p* < 0.01 with minimum voxel clusters of 10 contiguous voxels empirically chosen to maintain minimal type I and type II errors [74]. Because the objective of this study was to evaluate impact of fearful distractor on brain WM processing, a two-sample *t*-test was performed between groups voxelwise within ROIs and corrected for multiple comparison using the small volume correction approach in SPM [75]. *t*-tests were performed for high-loading fearful or neutral distractor phase as well as for low-loading fearful/neutral distractors. To determine whether behavioural performance influenced changes in BOLD activity between groups, mean percent signal change in BOLD activity extracted from voxels of significant group differences was correlated to performance accuracy and RT using Pearson correlation analysis controlling for age. Because the MCI group scored ~20% lower in MoCA test compared to HC, a Pearson correlation analysis was also performed to explore the relationship between BOLD signal within ROIs and MoCA scores.

## 3. Results

### 3.1. Behavioral Performance

The behavioural results of the WM task are presented in Table 2. Accuracy for the low-loading (2-letter) and high-loading (4-letter) condition was above 85% in both groups and in both fearful and neutral condition. Mixed model ANOVA did not identify any significant effect of group [*F*(1,279) = 1.72; *p* = 0.204], emotion [*F*(1,59) = 1.87; *p* = 0.186], or loading [*F*(1,24.59) = 0.294; *p* = 0.594] on response accuracy. Mixed model ANOVA was also performed on reaction time (RT) data. There was an effect of loading on RT in both groups [*F*(1,750667.55) = 126.571; *p* < 0.0001] but no effect of group [*F*(1,11708.238) = 1.974; *p* = 0.175] or emotion [*F*(1,1358.035) = 0.139; *p* = 0.713] and no interaction between group, load, and emotion.

Signal detection sensitivity was calculated for hits and false alarms. HC were found to have a hit rate of 100% for “yes” responses and 99.4% for “no” responses, while MCI had a 96.9% hit rate for “yes” responses and 98.6% for “no” responses. This translated to an approximated discriminability index (*d*′) of 5.15165 for HC and 3.7325 for MCI in regard to “yes” responses. We took this into account in image analysis by including only correct response trials.

### 3.2. Imaging Results

**Figure 2:**
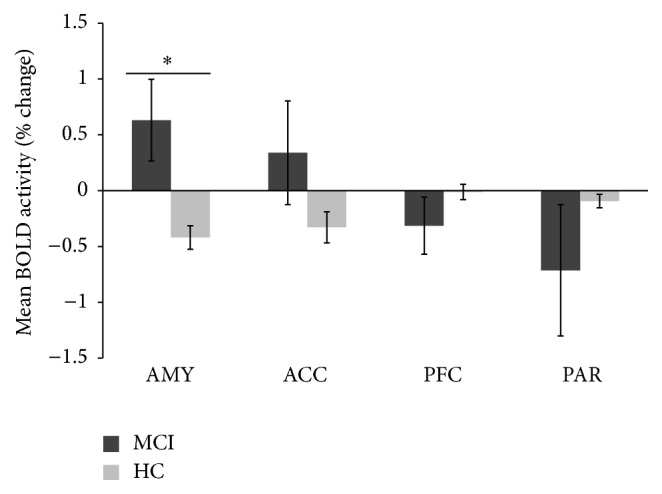
Plots of mean percent change in brain activity within* a priori* regions of interest in response to high-loading WM task during fearful-face distractors in MCI and HC subjects. Group mean % signal change average over all voxels within regions defined* a priori* from anatomical brain masks. Significant group differences were observed in the amygdala (AMY) (^*∗*^ *p* < 0.05). ACC: anterior cingulate cortex; PFC: prefrontal cortex; PAR: superior parietal gyrus.

Group means of percent change in BOLD activity for each of the ROIs are presented in Figure 2. Overall, the MCI group demonstrated an increase in brain activation in the anterior cingulate and amygdala area as compared to HC, while a decrease in brain activation was seen in the superior parietal and prefrontal areas in MCI patients. This finding is consistent with increased representation of emotional stimuli in MCI participants. The inverse correlation between higher amygdala activation and lower prefrontal and superior parietal areas has been reported by others [76].

Within the ROIs, a main effect of loading was found in the left precuneus (BA7), superior parietal cortex, and right anterior cingulate cortex (BA 32/24) (see Table 3). Likewise, significant group × loading × emotion interactions were observed in the anterior cingulate and medial and superior frontal gyri, as outlined in Table 3. Within groups, no significant differences between low loading and high loading were observed in ROIs (*p* < 0.01). When groups were compared on the low-loading condition (2-letter) data, we did not identify any significant differences in* a priori* selected ROIs, which was likely due to low processing demand. For the high-loading (4-letter) WM task with neutral distractor conditions, no significant difference between groups was observed.

#### 3.2.1. Differences in Activation during WM Fearful Task (F) between Two Groups

**Figure 3:**
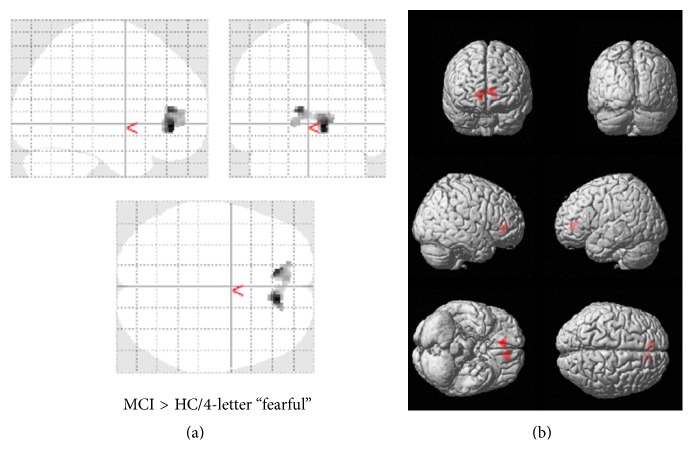
Areas of increased activation in MCI compared to HC for fearful-face high-load WM tasks. Regions showing MCI > HC in WM task with fearful-face distractor high-loading task. Contrasts are overlaid on a single subject image represented in 2 dimensions on a glass brain (b) and rendered in 3 dimensions on the cortex of the brain (a). Areas are listed in Table 4.

Focusing on the high-load (4-letter) WM task with fearful-face distractor, MCI patients showed higher activation than HC group in the* a priori* identified ROIs including the bilateral rACC (BA 32) and right medial frontal gyrus (BA 10) as outlined in Table 4 and shown in Figure 3. There was no increased brain activity in any of the brain areas of interest in the HC compared to MCI.

**Figure 4:**
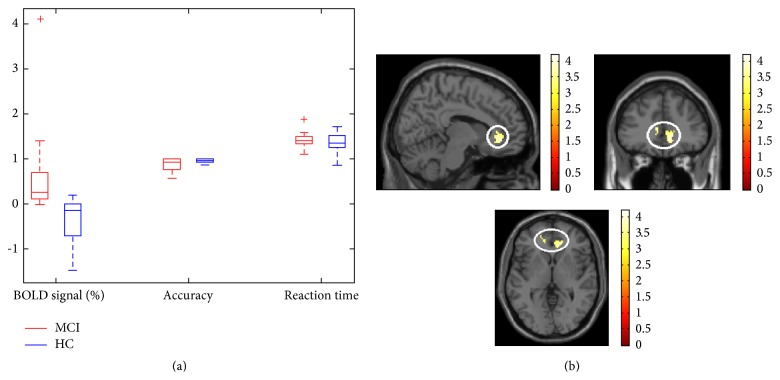
Plot of performance (accuracy) and mean change in brain activity in response to high-loading WM task during fearful-face condition. The mean percent change in BOLD signal was extracted from voxels significantly different between groups (bilateral ACC and right MFG, white circle). Accuracy and reaction time were normalized by an arbitrary reference value of 100 and 1000, respectively, to allow a single plot of all three variables. Although accuracy and reaction time were relatively equivalent between groups, the MCI patients had relatively higher change in BOLD response compared to controls. Images are displayed in radiological convention, which means the right side of the image corresponds to the left side of the brain.

The mean change in BOLD activity for each subject extracted from voxels of increased activity in MCI subjects compared to HC as listed in Table 4 was correlated to behavioural data. These voxels fell within regions in the bilateral ACC and right medial prefrontal cortex. An examination of the Mahalanobis distance and Cook's distance scores, a robust check for multivariate outliers, identified outliers in the data of MCI group; this was confirmed in boxplots (Figure 4). An extreme increase in BOLD activity was observed in one MCI subject, although the accuracy, RT, and MoCA scores of this subject were well within the sample's response range. In another MCI subject, a relatively longer RT was observed while BOLD activity, accuracy, and MoCA scores were within sample's range. No outlier was identified in HC data. When outliers were removed, the coefficients for associations of accuracy, RT, and MoCA scores with mean BOLD response in MCI were *r* = −0.85(8), *p* = 0.008; *r* = −0.42(7), *p* = 0.35; and *r* = −0.39(8), *p* = 0.37, respectively, and in HC were *r* = −0.06(12), *p* = 0.86; *r* = 0.015(12), *p* = 0.97; and *r* = −0.04(9), *p* = 0.93, respectively. The increased mean BOLD response in MCI compared to HC was maintained even after removal of outliers (*t*(18) = 3.22, *p* = 0.005). Together, these results indicate that, as performance declined, activation within ROIs in the MCI participants increased.

## 4. Discussion

The purpose of this study was to examine the effects of task-irrelevant emotional face stimuli on brain activity during a WM task in MCI patients. For this purpose, we used standardized fearful-face distractors that have been shown to reliably activate emotion-related brain areas. To the best of our knowledge, this is the first study to impose such task-irrelevant distractors during a WM task in MCI patients. This approach helps delineate the interaction between emotional facial expressions and attentional processing in this population, which evidence suggests is predisposed to negative cognitive biases and affective disorders. We tested the hypothesis that MCI is associated with a reduced capacity to regulate the impact of task-irrelevant emotional information on brain function. The results of this study support our prediction that adding fearful emotional distractors would result in a significantly higher level of activation in emotion-attention related areas in MCI patients compared to HC. Areas of significant difference included bottom-up “hot” areas of emotional processing, mainly the amygdala, and the ACC.

Although performance on the WM task did not differ significantly between groups and across conditions, our data suggest inefficiency in processing the task by the MCI group compared to HC. Overall, higher loading of the WM task (4 letters compared to 2 letters) resulted in increased activation in the precuneus, superior parietal, and anterior cingulate areas. These areas are reported to be involved in compensation for increased task demands with aging and in MCI [20, 41, 77]. On the other hand, and despite relatively equivalent performance, we found a robust correlation between performance accuracy on the WM task and BOLD signal in ROI during the fearful-face high-loading condition in the MCI group but not in HC. This suggests a compensatory mechanism required to perform the task in the MCI group when facing increased demands from the emotional stimulus.

When comparing MCI to HC based on our* a priori* hypothesis in ROI during the most demanding task (fearful high-loading condition), two areas show significant differences in BOLD signal: the ACC (BA 32 and BA 24) and medial frontal areas (BA 10). The ACC is believed to be part of an executive network involved in modulating cognition (WM) and emotions which has been implicated in dual-task working memory performance [65] and has been described as a compensatory mechanism for dealing with additional task demands [77]. Rostral ACC has also been implicated in resolving response conflict generated by emotional stimuli in healthy adults [37]. In our study, MCI patients performed equivalently to the HC group on the WM task with fearful-face distractors but showed increased activity in the amygdala, potentially reflecting increased encoding of negative distractors. Though speculative, one possibility is that the enhanced ACC (especially BA 32) activity observed in the MCI patient group may reflect inefficient regulatory influences of the rostral ACC over amygdala output. A similar kind of inefficiency of regulatory mechanisms has been demonstrated in patients with major depressive disorder [78]. However, it should be noted that our MCI patients did not have significant depressive symptoms. These results are consistent with the idea that emotional stimuli such as fearful-face distractors add demand on brain networks involved in executive-emotion tasks and that MCI state is associated with a compensatory mechanism to maintain the task in the face of an underlying pathology.

In our study, we found higher activation in the medial frontal area (BA 10) in MCI participants. This area has been implicated in multitasking, anticipation, social judgment, and understanding others' emotional state [79–81]. The multitasking is involved in maintaining the WM task while processing the fearful face likely drives activation of BA 10.

Our MCI participants' performance on the WM task was relatively equivalent to HC including accuracy, RT, and hit rate. This issue is important because it has generated concern in previous fMRI studies comparing MCI to HC during episodic memory encoding. Having equivalent behavioral performance strengthens the conclusions regarding group differences in brain activation.

Our study differed from the study by Döhnel et al. (2008) in that we utilized* task-irrelevant* emotional interference intersecting with a delayed matching WM task (as opposed to using emotional material as a* target* for the *n*-back WM task). Our study also differed from the study by Berger et al. (2015) in that we used fearful-face distractors instead of the IAPS as a* task-irrelevant* emotional distractor, allowing us to specifically explore the effect of facial emotional expression on WM processing. In our study, fearful-face stimuli activated the amygdala as predicted and likely resulted in bottom-up competition for attentional resources.

The prevalence of emotional symptoms, specifically anxiety, is high in MCI patients. The results of this study are important for considering the impact of emotional stimuli on WM function in MCI, which may in turn impact executive functioning in this group. This also highlights the importance of considering emotional stimuli when investigating WM in this population, an aspect that has been understudied.

There are important limitations that should be noted. We recruited our MCI participants from a memory clinic where patients present with memory concern and were classified as being in the amnestic MCI stage based on clinical criteria as described above. Although these patients were screened with global cognitive testing, no detailed neuropsychological battery results were available to further characterize the sample in terms of subtype of MCI (single-domain or multidomain amnestic). Also, we did not screen our participants for other neuropsychiatric symptoms that can affect emotional processing such as alexithymia or apathy. Alexithymia, which refers to impaired ability to identify and express one's emotions, was found to influence processing of negative emotions, predominantly in male participants and mainly as related to the processing of angry and sad facial expressions [82, 83]. In our study, we recruited only female participants and used only fearful-face expressions, both of which may reduce the impact of this confounder; however, whether our findings are gender specific or can be generalized across genders remains an important limitation to consider in future studies. Apathy also may affect emotional processing and is known to be a predictor for conversion to AD [84]. Therefore, future studies should consider screening and measuring apathy.

It is important to note that we deliberately restricted participation to females to avoid unanticipated gender differences in brain activation during cognitive-emotional tasks.

Our sample size is relatively modest due to the challenges in recruiting from this clinical sample. On the other hand, our sample size compares well to other influential studies in this area, including the study by Bokde et al. (2010) that recruited 8 MCI and 8 HC of mixed gender [15]. We were able to complete analysis on 9 MCI and 12 HC all of the same gender (females), which reduces variability in emotional processing and performance [61]. Future work with a larger sample, with higher level of WM task difficulty, and with different emotional face expressions would allow better understanding of the effect of emotional face interference on WM task processing.

Although we were able to detect BOLD signal difference as a result of the fearful-face distractor mainly in the high-loading condition, the time that our distractor was displayed was relatively short (1 second), in comparison with previous studies in young volunteers (e.g., see [85]). This may limit the impact of our emotional distractor, especially with the reported perceptual speed decreases with age [86] and its impact on the dedifferentiation process in response to sensory inputs [87]. This issue needs to be taken into account when designing future studies. Although the use of fearful-face distractor is justified in our study, other negative emotions such as anger and sadness need to be considered in future studies to ascertain whether the effect on brain activation is specific to fearful face or generalized to negative emotions in general.

## 5. Conclusion

To summarize, this study provides evidence to demonstrate that, compared to HC, female amnestic MCI patients show differential patterns of brain activation when performing a WM task in the presence of task-irrelevant fearful-face emotional distractors. These findings add insight into the role of emotional stimuli in modifying brain activity during WM tasks by adding burden to the task from bottom-up neuronal networks requiring higher levels of activation in areas involved in emotion-cognition integration. Future studies should include a larger sample size and both genders to allow better generalization. More detailed neuropsychological profiling of the MCI sample would help distinguish those with pure amnestic MCI from other types of MCI like nonamnestic and multidomain MCI and would allow exploration of cognitive domain effect on BOLD signal during emotion-WM interaction. Also, exploring the effect of other negative emotions like anger and sadness would clarify whether the effect we identified is specific to fearful face or is generalizable to other negative affective expressions.

## Figures and Tables

**Table 1:** Participants demographic and clinical data.

	MCI		HC		Comments
	Mean	SD	Mean	SD	
Age	72.7	9.3	65.8	6.5	ns (*p* = 0.066)
Education	10.5	0.8	10.7	2.2	ns
GDS-15	2.6	2.7	2	1.8	ns
MoCA	22.2	2.5	26.7	1.8	*p* < 0.001
Meds^*∗*^	1.8	0.78	1.5	0.8	ns
CDR^*∗∗*^	0.5		0		Clinical classification

SD: standard deviation; GDS: geriatric depression scale, 15 items; MoCA: Montreal Cognitive Assessment; ^*∗*^CDR: Clinical Dementia Rating Scale (a score of 0 indicates normal status, 0.5 indicates MCI status, and 1–3 indicate mild, moderate, and severe dementia (Morris, 1993 [67])). ^*∗∗*^Meds were prescribed by family physician and included antihypertensive and lipid lowering agents, small dose acetyl salicylic acid, and vitamins; one participant from each group was on small dose selective serotonin reuptake inhibitor for “minor mood symptoms,” and none of the participants were on cognitive enhancers. Comparisons were made using *t*-test, 2-tailed, 2-sample unequal variance.

**Table 2:** Summary of behavioral data related to accuracy of response and reaction time (RT).

Condition	Performance	MCI		HC	
		Mean	SD	Mean	SD
4-letter fearful	Accuracy	87.9%	14.7	95.2%	5.6
	RT	1450	204	1357	239

4-letter neutral	Accuracy	90.7%	10.7	93.5%	7.1
	RT	1456	169	1357	270

2-letter fearful	Accuracy	92.9%	13.1	92.9%	13
	RT	1304	281	1153	222

2-letter neutral	Accuracy	90%	9.7	96%	4.9
	RT	1277	219	1144	218

Data was expressed in percentage of accurate responses and milliseconds for RT. A mixed model ANOVA did not identify any significant effects of group, emotion, or loading on accuracy of response while RT was affected only with loading in both groups but with no significant effect of group or emotion.

**Table 3:** Summary of findings for interactions of loading and emotion between groups.

		MNI coordinates (*XYZ*)		Side	Area	Bdmn	*k*	*F* value	*p* value
Main effect of loading									
1	−12	−46	52	L	Precuneus	7	570	20.41	<0.0001
	−6	−50	48	L	Precuneus	7		14.07	<0.0001
	16	−44	54	R	Precuneus	7		12.21	0.001
2	30	−50	44	R	Superior parietal	7	50	10.36	0.002
3	10	42	6	R	Anterior cingulate	32	71	8.97	0.004
	2	36	2	R	Anterior cingulate	24		8.07	0.006

Interaction: group by loading									
1	50	2	36	R	Precentral gyrus	6	12	10.24	0.002
2	−14	50	−2	L	Anterior cingulate	10	28	8.33	0.005

Interaction: group by emotion									
1	−8	36	8	L	Anterior cingulate	32	23	12.22	0.001
2	48	42	30	R	Middle frontal gyrus	46	17	10.67	0.002
3	−28	54	34	L	Superior frontal	9	60	10.01	0.002

Interaction: group by loading by emotion									
1	12	38	−4	R	Medial frontal gyrus	10	48	10.44	0.002
2	18	52	14	R	Superior frontal	10	61	9.70	0.003
	12	48	22	R	Superior frontal	9		8.59	0.004
	22	42	20	R	Superior frontal	10		7.43	0.008
3	−12	42	0	L	Anterior cingulate	*∗*	37	8.69	0.004
	−14	52	2	L	Medial frontal gyrus	10		8.40	0.005

Brain areas and corresponding Brodmann (Bdmn) regions are listed for activation peaks at significance level of minimum 10 voxels per cluster (*k*), *p* < 0.01, for each between-group analysis. L: left; R: right.

**Table 4:** Brain regions of increased activation in MCI patients compared to HC during WM high-loading response to fearful face.

Region	Side	Brodmann area	MNI coordinates (*XYZ*)	*T*-value
Medial frontal	R	10	12; 38; −4	4.15
Anterior cingulate	L	32	−10; 38; −8	3.90
Anterior cingulate	R	32	14; 36; 0	3.86
Anterior cingulate	L	32	−6; −46; 6	3.44
Anterior cingulate	L	10	−16; 48; −2	*3.43*
Anterior cingulate	R	32	20; 42; 2	*3.41*
Anterior cingulate	L		−10; 40; 0	*3.39*
Anterior cingulate	L	32	−14; 44; −4	*3.34*
Anterior cingulate	L	24	−8; 36; 2	*3.31*
Anterior cingulate	L	32	0; 44; 6	*3.29*
Anterior cingulate	R	32	4; 44; 4	*3.29*

Brain regions and corresponding Brodmann areas are listed from a cluster of 241 voxels, corrected using small volume correction, *p* = 0.015, FWE (familywise error). Regions are displayed in Figure 2.
